# Radio Electric Asymmetric Conveyer Tissue Reparative Treatment on Post-surgical Breast Skin Necrosis. A Report of Four Cases

**DOI:** 10.7759/cureus.25666

**Published:** 2022-06-05

**Authors:** Vania Fontani, José Alfredo Coelho Pereira, Salvatore Rinaldi

**Affiliations:** 1 Research, Rinaldi Fontani Foundation, Florence, ITA; 2 Regenerative Medicine, Rinaldi Fontani Institute, Florence, ITA

**Keywords:** regenerative medicine therapies, reparative medicine, breast, reac, biostimulation, wound care, plastic and reconstructive surgery, nac necrosis

## Abstract

Breast surgical treatments for both tumors and aesthetic reasons are very frequent. The nipple-areola complex (NAC) ischemia is a possible complication after breast surgery. This lesion can be devastating for the patient in the post-surgical course and can lead to final epidermolysis. The necrosis is generally attributed to vascular compromise or excessive tension of the flaps. Actually, the phenomena that prevent spontaneous repair are due to variations in the endogenous electrical potential at the cellular level. In damaged tissues, the electric potential difference across the epithelium is often profoundly altered.

In this manuscript, we are presenting four cases of NAC necrosis that were successfully treated with reparative tissue optimization (TO-RPR) treatment of the Radio Electric Asymmetric Conveyer (REAC) technology.

REAC technology was conceived to overcome the limits of exogenous electrical stimulations. Instead of administering an electrical stimulus that imposes itself on the endogenous bioelectric activity (EBA), the REAC technology restores the correct potential difference inside the tissues, which is essential for all reparative and regenerative processes. The REAC treatment applied was able to promote a fast-healing process of the necrosis of the NAC following surgery of the breast.

## Introduction

In breast surgery, tissue necrosis is due to vascular compromise or excessive tension of the flaps. The area in the process of necrosis takes on a bluish-purplish color and subsequently toward pallor and the final epidermolysis. In general, this complication is infrequent in cosmetic breast surgery, but nipple-areola complex (NAC) ischemia occurs in up to 15% of cases [1].

Although the causes that can determine ischemic areas and tissue necrosis in post-breast surgery can be easily recognized in vascular compromise or excessive tension of the flaps, the phenomena that prevent spontaneous repair are due to variations in the endogenous electrical potential [2,3]. Actually, in healthy tissues an electric potential difference across the epithelium is maintained, while this potential difference in damaged tissues is often profoundly altered, making it more difficult or even preventing the reparative processes [3]. Various techniques of exogenous electrical stimulation have been proposed to restore the endogenous electrical potential, and recover and promote the directional migration of cells and signaling molecules through electrotaxis [4]. The Radio Electric Asymmetric Conveyer (REAC) technology was conceived to overcome the limits of exogenous electrical stimulations. In fact, instead of administering an electrical stimulus that imposes itself on the endogenous bioelectric activity (EBA), the REAC technology induces inside the tissues, even deep ones, a potential difference that favors the restoration of the correct EBA, fundamental for all reparative and regenerative processes [2].

## Case presentation

Case 1

**Figure 1:**
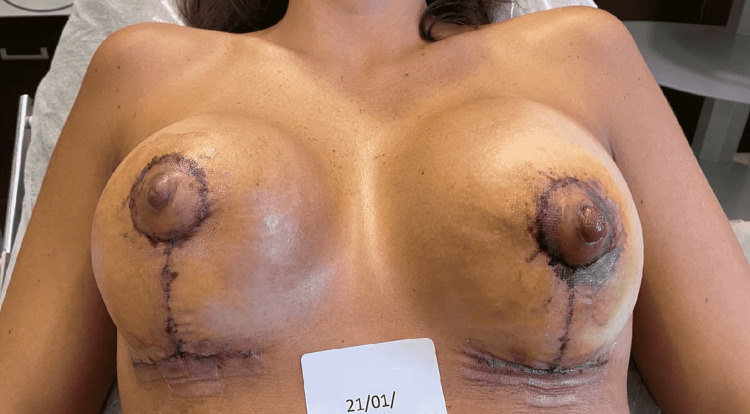
Situation of the left nipple-areola complex (NAC) after two days from surgery. The signs of post-surgical epidermolysis begin to be evident.

The first case is a 35-year-old female patient, who underwent breast augmentation surgery. After two days, the follow-up visit revealed post-surgical epidermolysis of the NAC. Epidermolysis is a skin alteration in which, for even mild stimuli, a detachment takes place between cellular elements of the same layer or different layers of the epidermis. The process usually takes place in the first two days and if the patient is blindfolded it becomes difficult to notice the event. The surgeon advised the patient to undergo a cycle of REAC reparative tissue optimization (TO-RPR) treatments in order to promote NAC healing. The patient came to our observation on January 21 and started the REAC TO-RPR treatment on the same day (Figure 1).

**Figure 2:**
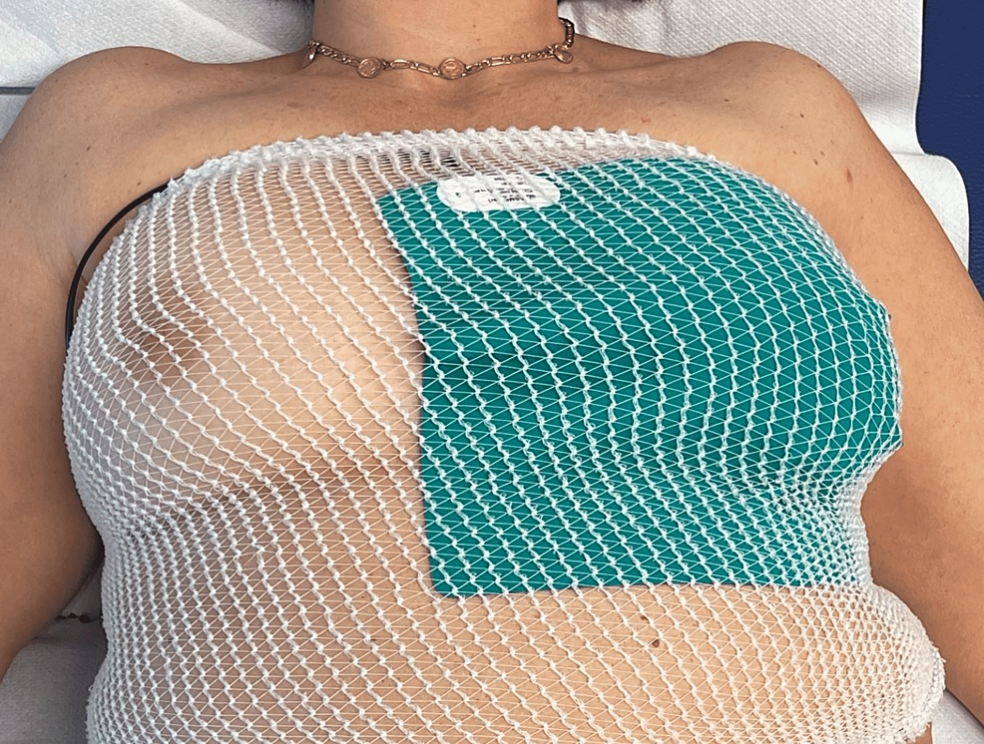
Example of administration of the TO-RPR treatment.

Each REAC TO-RPR treatment session lasted 15 minutes, according to the standardized procedure. The treatment parameters are preprogrammed and cannot be changed by the operator. The treatment is administered by placing an asymmetric conveyer probe (ACP) on the area to be treated. The ACP is kept on the area by means of a tubular elastic gauze (Figure 2) and connected to the REAC device, BENE Model 110 (therapeutic electromedical equipment for neurobiological stimulation CE 1282; ASMED SRL, Florence, Italy).

**Figure 3:**
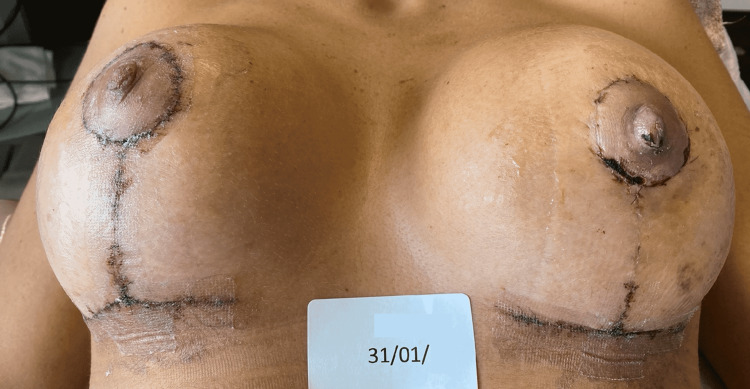
Situation of the NAC immediately after the administration of the 18th REAC TO-RPR treatment session. NAC - nipple-areola complex; REAC - Radio Electric Asymmetric Conveyer

The REAC TO-RPR cycle was administered three sessions per day for six days for a total of 18 sessions. At the end of the 18th REAC TO-RPR treatment session, the patient showed nearly complete healing of her NAC lesion (Figure 3).

Case 2

**Figure 4:**
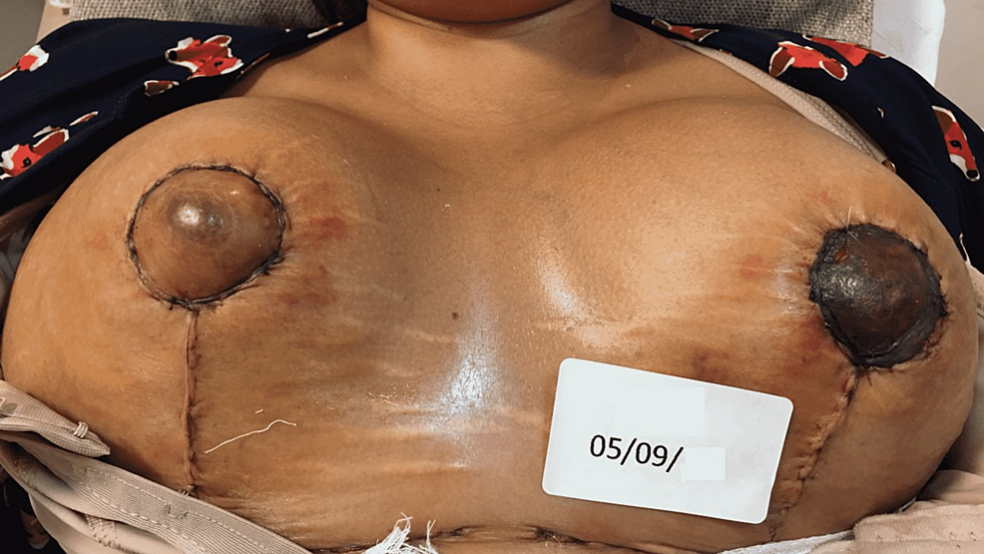
Situation of the left NAC after two days from surgery: the signs of post-surgical epidermolysis begin to be evident. NAC - nipple-areola complex

The second case is a 44-year-old female patient, a heavy smoker, who did not quit smoking either before or after breast surgery. After two days, the follow-up visit revealed post-surgical epidermolysis of the NAC (Figure 4).

The surgeon advised the patient to undergo a cycle of REAC TO-RPR treatments in order to promote the NAC healing. The 15 minutes REAC TO-RPR treatment was administered once a day, over a period of approximately 20 days.

**Figure 5:**
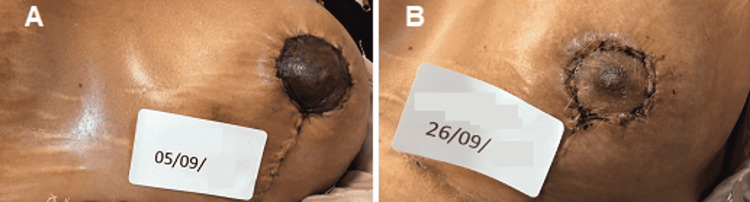
NAC lesion before (A) and immediately after (B) the 18th session of the REAC TO-RPR treatment. NAC - nipple-areola complex; REAC - Radio Electric Asymmetric Conveyer

At the end of the 18th REAC TO-RPR treatment session, the comparison between the NAC lesion before (Figure 5A) and after (Figure 5B) REAC TO-RPR treatment showed nearly complete healing.

Case 3

**Figure 6:**
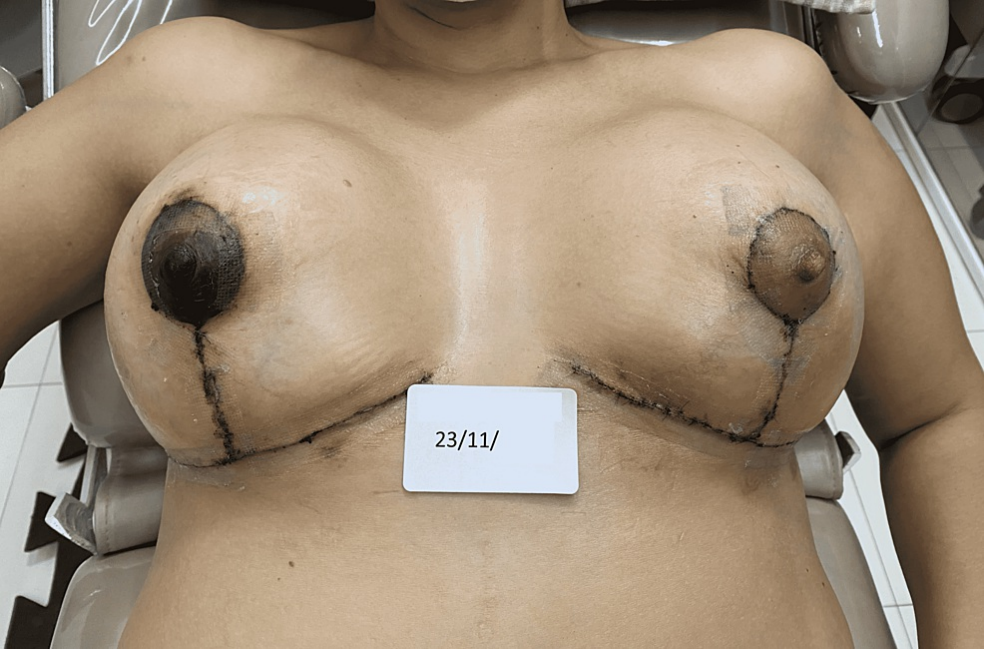
Situation of the right NAC after three days of surgery: the signs of post-surgical NAC necrosis begin to be evident. NAC - nipple-areola complex

The third case is a 43-year-old female patient, with a history of obesity and undergoing bariatric surgery, who underwent a second mastopexy surgery, with post-surgical epidermolysis of the NAC in the first 24 hours. The surgeon advised the patient to undergo a cycle of REAC TO-RPR treatment to promote the NAC healing. The patient came to our observation on November 23 (Figure 6) and started the REAC TO-RPR treatment on the same day.

**Figure 7:**
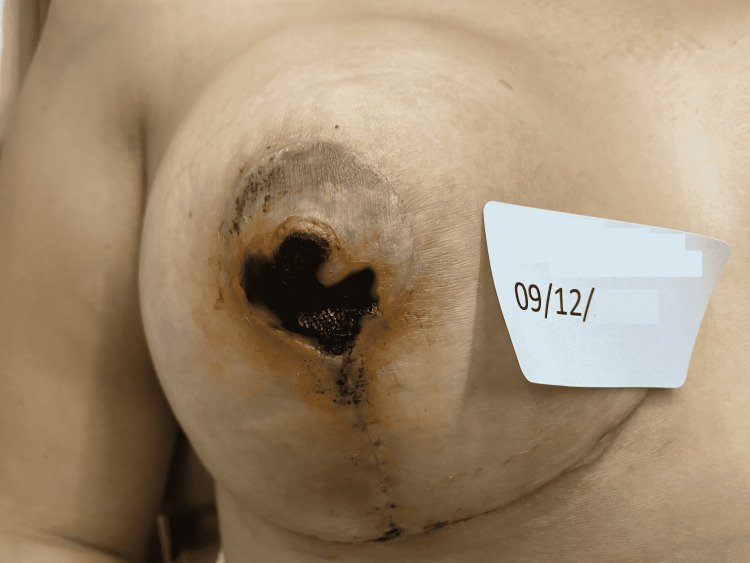
The NAC immediately after the 18th session of the REAC TO-RPR treatment. NAC - nipple-areola complex; REAC - Radio Electric Asymmetric Conveyer

The 15 minutes REAC TO-RPR treatment was administered once a day, over a period of approximately 20 days. At the end of the 18th REAC TO-RPR treatment session, the patient showed nearly complete healing of her NAC lesion (Figure 7).

**Figure 8:**
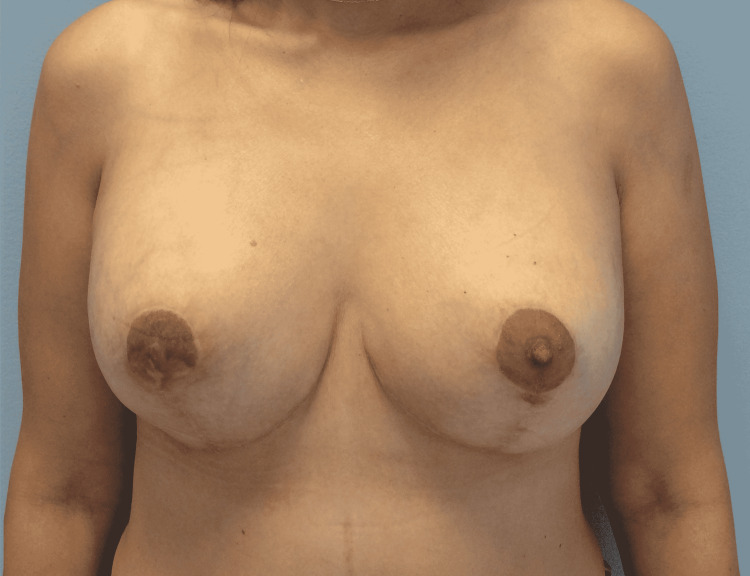
The NAC one month after the last REAC TO-RPR treatment session. NAC - nipple-areola complex; REAC - Radio Electric Asymmetric Conveyer

A further improvement was observed after one month from the end of the last REAC TO-RPR treatment (Figure 8).

Case 4

**Figure 9:**
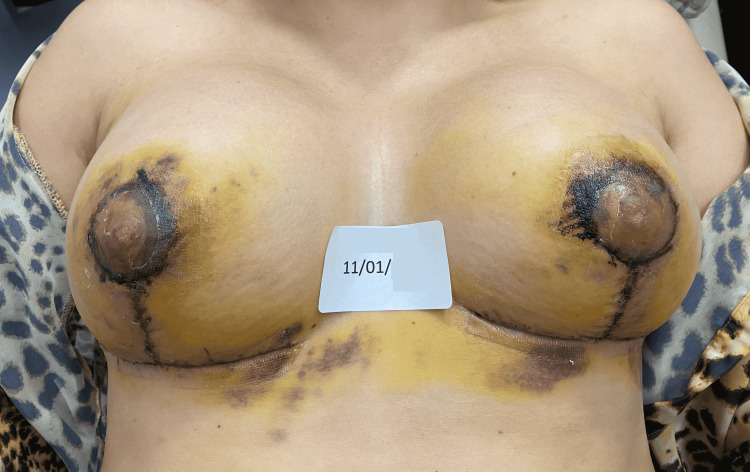
Situation of the NACs after three days of surgery: the signs of post-surgical NAC necrosis begin to be evident. NAC - nipple-areola complex

The fourth case is a 34-year-old post-pregnancy patient, with weight gain above the expected, associated with breast enlargement. In the first 48 hours after the surgery, the patient presented bilateral post-surgical epidermolysis of the NAC. The patient came to our observation on January 11, starting the REAC TO-RPR treatment on the same day (Figure 9).

**Figure 10:**
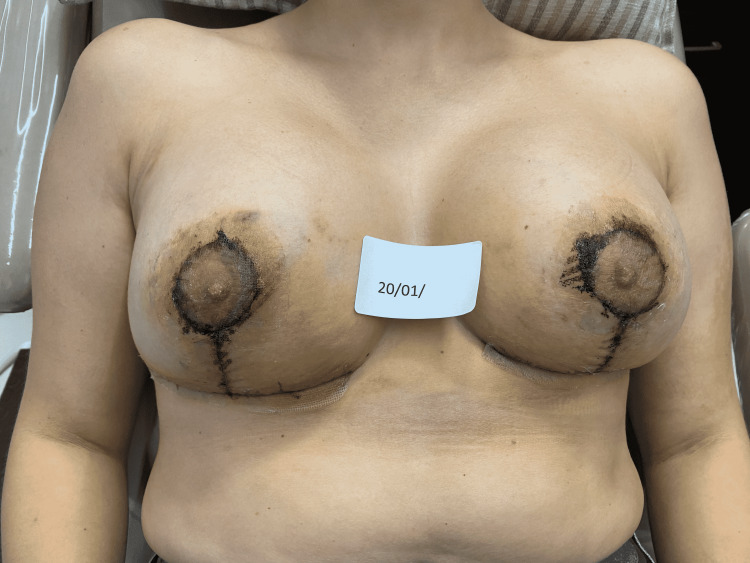
The NACs immediately after the 18th REAC TO-RPR treatment session. NAC - nipple-areola complex; REAC - Radio Electric Asymmetric Conveyer

The 15 minutes REAC TO-RPR treatment was administered once a day, over a period of approximately 20 days. On January 20, at the end of the 18th REAC TO-RPR treatment session, the patient showed nearly complete healing of her NAC lesions (Figure 10).

**Figure 11:**
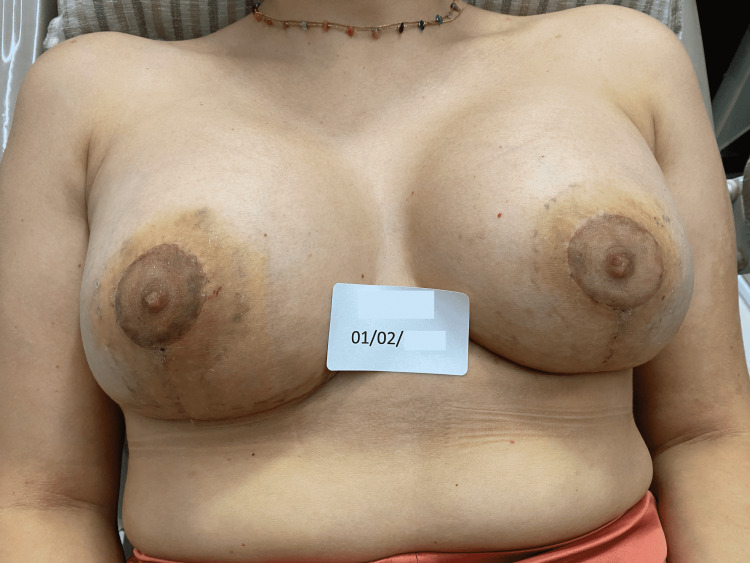
The NACs 20 days after the last REAC TO-RPR treatment session. NAC - nipple-areola complex; REAC - Radio Electric Asymmetric Conveyer

A further improvement was observed after one month from the end of the last REAC TO-RPR treatment (Figure 11).

## Discussion

Breast surgical treatments for both tumors and aesthetic reasons are very frequent. As a consequence of these interventions, ischemic suffering of NAC sometimes occurs. This unexpected event can seriously compromise the result of the surgery, but above all, it can determine a serious dissatisfaction in the patient, as an NAC lesion is a complication that can be devastating in the post-surgical course of breast surgery.

For this reason, surgical techniques have evolved to reduce the possibility of this complication [5,6]. Despite the refinement of surgical techniques, a percentage of NAC lesions, which varies according to the various studies, is always present [7]. These complication rates can increase in women who have been smokers for a long time [8].

In addition to re-intervening surgically, other approaches have been proposed to promote the healing of post-surgical necrosis of NAC. Among these, are the use of advanced dressings which can be silver-based to reduce bacterial burden, vacuum dressing [9], or hyperbaric oxygen therapy (HOT) [10].

One of the main limitations of HOT is the need to have a hyperbaric oxygen chamber. Furthermore, the treatment has a duration of about 90 minutes at 2.0 atmospheres for about 30 sessions. Beyond these techniques, the use of various electrostimulation technologies to promote skin tissue repair is increasingly emerging [3].

REAC technology is an example of neurobiological stimulation applied to promote tissue repair. The recommended treatment protocol is the post-surgical wound dressing, which during the 18 sessions of REAC TO-RPR is momentarily removed to allow contact of the APC to the skin.

The REAC peculiar mechanism of action does not consist in the administration of an exogenous current, but it determines the progressive restoration of the asymmetries of electrical charges in tissues [11-13]. This phenomenon allows the recovery of ionic flows and therefore of the cellular EBA, which is essential to activate the fine reparative processes of the tissues [11-13].

The REAC functional recovery of the correct EBA is able to promote reparative [11,14,15] and regenerative processes [16-18], up to determining phenomena of direct cellular reprogramming [12,13,19,20]. The possibility of obtaining refined reparative processes in a short time is particularly useful when it is desirable to achieve results that are also aesthetically pleasing, such as in the cases reported in this manuscript.

## Conclusions

The results presented in this manuscript highlight how one of the least desired complications of breast surgery, such as NAC necrosis, can now have new therapeutic approaches of neurobiological stimulation, aimed at repairing such a complex and delicate part of the body, as NAC.

The REAC TO-RPR treatment is a non-invasive and painless treatment of easy and fast administration, able to promote a fast-healing process of the necrosis of the NAC following surgery of the breast. Due to these characteristics, the REAC TO-RPR could represent a valid treatment not only in post-surgery but also for prevention to reduce the risk of NAC lesions.
